# Integrated Multiomics Validation of Key *MUC* Gene Expression for the Signature Biomarker in the Pakistani Cohort

**DOI:** 10.1155/bmri/9777346

**Published:** 2025-10-23

**Authors:** Maryam Naeem, Nimra Munir, Ibrar Ahmed, Zarrin Basharat, Aneesa Sultan

**Affiliations:** ^1^ Biochemistry Department, Quaid-e-Azam University, Islamabad, Pakistan, qau.edu.pk; ^2^ Alpha Genomics Private Limited, Islamabad, Pakistan; ^3^ Microbiological Analysis Team, Group for Biometrology, Korea Research Institute of Standards and Science (KRISS), Daejeon, Republic of Korea, kriss.re.kr

**Keywords:** in vitro validation, MAMs, noninvasive approach, PDAC, RT-qPCR, signature biomarker, transcriptome analysis, WES

## Abstract

**Purpose:**

Pancreatic ductal adenocarcinoma (PDAC) is the 3^rd^ leading cause of cancer‐related mortalities and has a poor prognosis, with a 5‐year survival of 8%–9%. The major challenge associated with management of PDAC is delayed diagnosis. Diagnosis at an early stage can significantly increase the survival. Therefore, we used multiomics analysis to validate a novel signature biomarker of early‐stage PDAC. The signature was derived from a computational analysis and subsequently validated in the Pakistani patient cohort, including patients with well‐defined early‐stage (I and II) PDAC.

**Methods:**

With the aim of validating the signature biomarker (*MUC3A*/*MUC4*/*MUC13*/*MUC16*) as a potent early diagnostic and prognostic marker, a comprehensive analysis of multiomics data of the Pakistani cohort was performed utilizing the integrated whole‐exome sequencing, transcriptome analysis, and RT‐qPCR.

**Results:**

The in vitro validation of a signature biomarker in the Pakistani cohort reveals the pathogenic effect of mutations in the key *MUC* genes and its profound effect on its protein functionality. Among all key *MUC* genes analyzed in a transcriptomic dataset, only MUC4 exhibits statistically significant differential expression. Finally, RT‐qPCR results demonstrate significant overexpression of all key *MUC* genes in PDAC blood samples.

**Conclusion:**

In conclusion, our findings suggest *MUC3A/MUC4/MUC13/MUC16* as robust diagnostic and prognostic biomarkers and liquid biopsy as a noninvasive approach to be integrated into daily clinical practice. This analysis can also be extended to other malignancies.

## 1. Introduction

Pancreatic cancer presents as a growing health concern, characterized by alarming morbidity and mortality rates [1]. Despite its relatively low incidence, pancreatic cancer ranks as the third leading cause of cancer‐related deaths [2]. Projections suggest it may rise to become the second leading cause of cancer‐related deaths in the United States by 2030 [3]. PDAC carries a dismal prognosis, ranking among the deadliest malignancies, with a 5‐year survival rate of only 8%–9% [4, 5]. At the time of diagnosis, a mere 20% of patients are eligible for surgical resection, while the remaining 80% present with unresectable, metastatic disease [1, 4, 6]. Early diagnosis, particularly at Stage IA, dramatically improves prognosis, with a 5‐year survival rate approaching 70% [7, 8]. This underscores the urgent need for reliable diagnostic biomarkers and innovative diagnostic approaches to enhance the management and treatment of this disease.

Mucins, high molecular weight O‐glycoproteins, are primarily involved in cell signaling, cell–cell junctions, strengthening barrier defense, and lubricating the surfaces of epithelial cells [9]. Based on their structure, mucins are categorized into membrane‐associated mucins (MAMs) and secreted/gel‐forming mucins [10, 11]. They are encoded by *MUC* genes and exhibit notably altered expression and glycosylation patterns in diverse cancers, particularly the cancers of epithelial origin, such as breast, pancreatic, colon, gastric, ovarian, lung, and biliary cancers [12]. In the disease‐free situation, MAMs typically exhibit minimal expression in the pancreas [13, 14].

In our previous study, the integrated computational analysis revealed differential expression of MAMs, namely, *MUC1*, *MUC3A*, *MUC4*, *MUC12*, *MUC13*, *MUC16*, *MUC17*, and *MUC20*. The expression profiling of overexpressed MAMs explored the cancer‐specific overexpression of *MUC3A*, *MUC4*, *MUC13*, and *MUC16* in tumor samples as compared to control. Based on the expression analysis, these genes were selected as key *MUC* genes. Specifically localized expression of key *MUC* genes in the ductal cell cluster of the pancreas demonstrated relatively low expression under normal conditions. For investigating the prognostic potential, the stage‐specific expression analysis was performed which disclosed the highest expression of key *MUC* genes at the earliest stage of cancer (IA, IB, IIA, and IIB). Moreover, the disease‐free survival (DFS) analysis reinforced its prognostic potential by suggesting the strong positive correlation between poor survival outcome and high expression of genes as signature. Furthermore, the coexpression analysis indicated that all key *MUC* genes were negatively correlated with each other in terms of their expression which strengthened its tendency as a multigene biomarker. Additionally, the top most correlated genes were subjected to the functional enrichment analysis to disclose their significant enrichment in the biological functions and pathways related to carcinogenesis. Finally, the PPI network was constructed to uncover the genes that were critically linked with key *MUC* genes.

To address these objectives, the present study advances our previous research through comprehensive in vitro validation of key *MUC* genes’ expression profile from the samples of the Pakistani cohort. The present study adopts multiomics modality, comprising whole‐exome sequencing (WES), transcriptome analysis (RNA sequencing [RNA‐seq]), and reverse transcription‐quantitative polymerase chain reaction (RT‐qPCR) to validate expression and evaluate mutational landscape of *MUC3A*, *MUC4*, *MUC13*, and *MUC16*. The study is aimed at validating the signature biomarker (*MUC3A*/*MUC4*/*MUC13*/*MUC16*), employing peripheral blood and tissue samples from the Pakistani cohort to ensure that the computational findings are relevant and accurate in a real‐world clinical scenario. This approach seeks to improve the diagnosis, treatment, and management of PDAC.

## 2. Material and Methods

### 2.1. Ethical Approval and Sample Collection

This study was approved by the Pakistan Institute of Medical Sciences (PIMS), Islamabad, under the reference number FMTI 20821. The samples were collected under the protocols approved by the ethics committee of Quaid‐e‐Azam University. Written informed consent was obtained from each patient before the collection of blood and tissue samples. A total of 6 blood samples from healthy individuals and 25 blood samples from PDAC patients are used in this study. Besides this, three tumor samples along with their three adjacent controls (*n* = 3 + 3) are used for exome sequencing and analysis.

The blood samples were collected in EDTA tubes and stored at −80°C for downstream processing. Tumor and control tissue samples were collected during the surgical procedure and immediately transferred in liquid nitrogen to be flash frozen and subsequently stored at −80°C for further extractions. Normal tissue samples were obtained from the areas of the pancreas that were confirmed noncancerous by a pathologist.

Detailed clinicopathological and demographic parameters of patients from whom PDAC blood samples were collected are mentioned in Table 1.

**Table 1:** Clinicopathological and demographic parameters of the Pakistani cohort.

**Sample status**	**AJCC stage**	**Sex**		**Age range**
		**Male**	**Female**	
PDAC	I	4	2	40–45
	II	0	0	
	I	2	2	46–50
	II	0	0	
	I	5	0	51–55
	II	1	0	
	I	0	1	56–60
	II	2	2	
	I	0	0	61–65
	II	2	2	

### 2.2. DNA Isolation and WES

Genomic DNA was extracted from tissue samples using the standard phenol‐chloroform protocol [15]. The DNA concentration and purity were assessed using a MultiSkan GO spectrophotometer (Thermo Scientific) [16]. WES was performed on an Illumina HiSeq platform [17]. The workflow began with base calling to generate raw reads in FastQ format, followed by quality assessment using FastQC and adaptor removal via FastP. The high‐quality reads were aligned to the human reference genome (GRCh38/hg38) using the Burrows–Wheeler Aligner (BWA) [18]. Postalignment processing involves marking of PCR duplicates using the MarkDuplicate tool. The GATK pipeline was utilized for variant calling [18, 19]. MuTect2 was employed to annotate somatic variations [20, 21]. ANNOVAR was used to functionally annotate the variations [22].

### 2.3. Mutational Profiling of Key *MUC* Genes

Mutational analysis was conducted focusing on the key *MUC* genes. The mutations were filtered based on their pathogenic potential in tumors. The analysis of target genes was based on meeting the following criteria: nonsynonymous alterations, disease causing, and being located within exonic or splice site regions [23].

### 2.4. RNA Isolation and Transcriptome‐Seq

Total RNA was isolated from tissue samples using the TRIzol method. The isolated RNA was quantified via the Thermo Scientific MultiSkan Go Instrument. Complementary DNA (cDNA) was synthesized by using the RevertAid Reverse Transcriptase cDNA synthesis kit (Thermo Fisher Scientific) [24]. Subsequently, transcriptome sequencing was performed on the Illumina sequencing platform. The resulting transcriptome sequencing data was analyzed using R programming, with bioinformatics tools for differential expression analysis [25].

### 2.5. RT‐qPCR

To validate the expression profile of the key *MUC* gene, RT‐qPCR was carried out on 18 pancreatic cancer patients’ blood samples. Quantitative PCR was performed using the Mic PCR (Bio Molecular System) with Syber Green detection. Primers for the target genes were designed using the PRIMER3 software (https://primer3.ut.ee/) to span exon–exon junctions, ensuring specificity [26]. The housekeeping gene GAPDH was optimized as an internal control [27]. All reactions were processed in duplicates. The relative mRNA level of key *MUC* genes was calculated by employing the 2^−*Δ* *Δ*Ct^ method [28].

Statistical analysis was carried out by using GraphPad Prism Version 10 for Windows (https://www.graphpad.com/). Differences in the expression level of key *MUC* genes across the tumor and control samples were analyzed using Student’s *t*‐test [29]. A *p*value < 0.001 was considered to be statistically significant [30].

Primer sequences are listed in Table 2.

**Table 2:** Primer sequences used for RT‐qPCR analysis, including forward (F) and reverse (R) sequences for each target gene.

**Sr. no**	**Primers**	**Tm**	**Amplicon size**	**Sequence**
1	*MUC3A-F*	59.6°C	102 bp	CCTTAGACGTCCTCCCCTTT
	*MUC3A-R*	59.7°C		GCTAGGGACCAGGATGTGAG
2	*MUC4-F*	59.7°C	94 bp	CAGACACACCCACAGAAGGA
	*MUC4-R*	59.3°C		ACTTAGGGCCATCACCACAT
3	*MUC13-F*	59.9°C	107 bp	CCAGCCTCTCTGAATGGAAG
	*MUC13-R*	59.7°C		TCTGACAAGCGCTAATAGCATC
4	*MUC16-F*	59.3°C	103 bp	ATGGTACCCAGCTGCAGAA
	*MUC16-R*	59.4°C		GGAAGGTCAGAATTCCCAGTT
5	*GAPDH-F*	58°C	106 bp	CAGCAAGAGCACAAGAGGAA
	*GAPDH-R*	59°C		TCTACATGGCAACTGTGAGGA

## 3. Results

### 3.1. Mutational Profiling of Key *MUC* Genes

**Figure 1:**
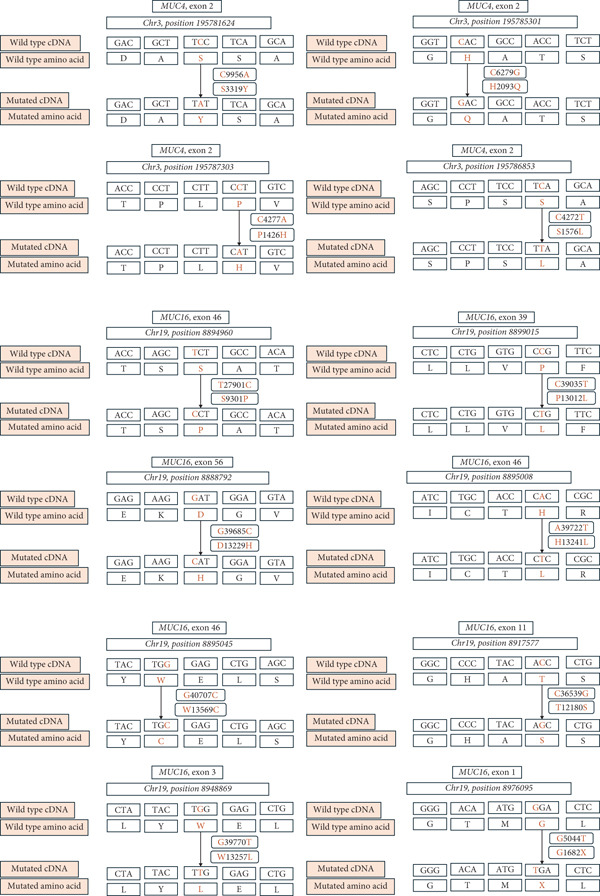
Schematic representation of pathogenic mutations in *MUC4* and *MUC16.*

**Figure 2:**
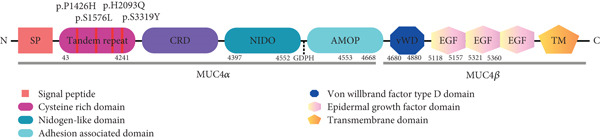
Schematic representation of the *MUC4* domain. This figure depicts the domain structure of the protein, highlighting the regions of the tandem repeat domain showcasing the sequence variability contributing to the functional diversity and interaction capabilities.

**Figure 3:**
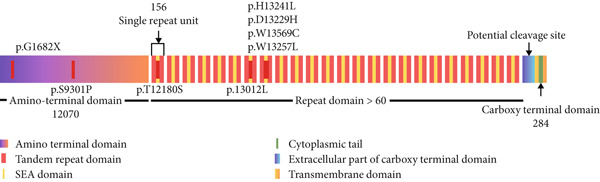
Schematic representation of the *MUC16* domain. This figure depicts the domain structure of the protein, highlighting the regions of the amino terminal and tandem repeat domain where the variation occurs.

After performing WES analysis in three matched tumor/normal pairs, a total of 2,581,210 variants were identified along with the number of variants in each sample, 5488 of which were nonsynonymous. Several novel mutations in *MUC3A*, *MUC4*, and *MUC16* were discovered except for *MUC13* (Supporting Information: SiAPC‐2‐somatic‐exonic‐xlsx, S7APC‐exonic.xlsx, and S8APC‐exonic‐xlsx). The pathogenicity of these variants was assessed by the following computational algorithms: Damagepredcount, SIFT [31], Polyphen2 [32], MutationTaster [33], FATHMM [34], VEST4 [35], PROVEAN [36], BAYESDel [37], ClinPred [38], and REVEL [39]. The score range was 0–1. The higher the score, the more deleterious the variant was. Following algorithmic application, detailed analysis of four variants in MUC4 and eight variants in MUC16 genes has been systematically organised, including particular nucleotide and amino acid alteration, exon numbers, and chromosomal positions (Figure 1). Four variants in MUC4 (Figure 2) and eight MUC16 (Figure 3) variants were identified as putatively pathogenic.

The summary of individual pathogenic mutations and associated protein coding change of *MUC4* and *MUC16* among all PDAC tissue samples is shown in Table 3.

**Table 3:** Highly deleterious mutations of MAMs identified in PDAC samples through WES.

**MAMs**	**Exon**	**HGVS_c.**	**HGVS_p**	**Mutation**	**Interpretation**	**Genotype**
*MUC4*	2	c.C9956A	p.S3319Y	Missense	Pathogenic	Heterozygous
*MUC4*	2	c.C6279G	p.H2093Q	Missense	Pathogenic	Heterozygous
*MUC4*	2	c.C4277A	p.P1426H	Missense	Pathogenic	Heterozygous
*MUC4*	2	c.C4727T	p.S1576L	Missense	Pathogenic	Heterozygous
*MUC16*	46	c.G39770T	p.W13257L	Missense	Pathogenic	Heterozygous
*MUC16*	39	c.C39035T	p.P13012L	Missense	Pathogenic	Heterozygous
*MUC16*	56	c.G40707C	p.W13569C	Missense	Pathogenic	Heterozygous
*MUC16*	46	c.A39722T	p.H13241L	Missense	Pathogenic	Heterozygous
*MUC16*	46	c.G39685C	p.D13229H	Missense	Pathogenic	Heterozygous
*MUC16*	11	c.C36539G	p.T12180S	Missense	Pathogenic	Heterozygous
*MUC16*	3	c.T27901C	p.S9301P	Missense	Pathogenic	Heterozygous
*MUC16*	1	c.G5044T	p.G1682X	Stopgain	Pathogenic	Heterozygous

Figure 1 shows that the missense mutations in MUC4 and MUC16 are expected to cause abnormal protein function, contributing to the neoplastic processes in PDAC.

The protein domain structure of *MUC4* (Figure 2) is depicted schematically, highlighting the different domains and the specific locations of amino acid variations. The tandem repeat domain is emphasized, showcasing the positions where amino acid substitutions occur.

This schematic representation of the *MUC16* domain structure (Figure 3) highlights the specific location of amino acid transitions. Most mutations are predominantly clustered within the tandem repeat domain region and amino terminal domain.

### 3.2. Transcriptomic Analysis of Key *MUC* Genes

**Figure 4:**
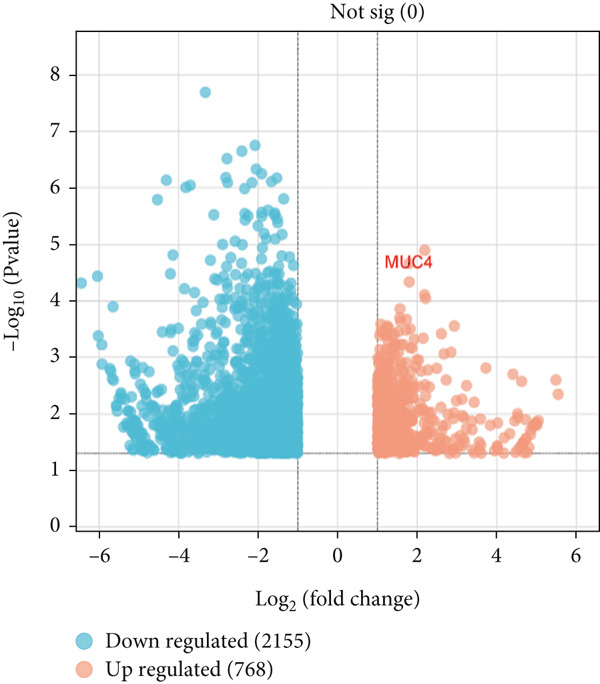
A volcano plot of differentially expressed genes identified through transcriptomic analysis of matched tumor and adjacent normal tissue samples from PDAC patients of the Pakistani population (*n* = 3 tumor tissues; *n* = 3 matched adjacent normal tissues). This volcano plot highlights *MUC4* which is significantly differentially expressed among the key *MUC* genes.

In the transcriptomic data, we used differential expression analysis to generate a volcano plot. This plot illustrates the relationship between the magnitude of fold change and the statistical significance of gene expression differences. Genes with large fold changes and low *p* values are highlighted, indicating significant differential expression. The parameters were established at |log2FC| > 1.5 and *p* < 0.05. There were 2923 DEGs found in all, with 768 genes being upregulated and 2155 genes being downregulated (Supporting Information: Significant.csv). The plot illustrates the most significantly upregulated *MUC4* (log2FC = 1.794 and *p* value = 2.17e − 5), marked on the plot. Notably, among the key *MUC* genes investigated, *MUC4* was the only one with statistically significant differential expression (Figure 4).

### 3.3. RT‐qPCR for Quantitative Validation of the Gene Expression Profile

**Figure 5:**
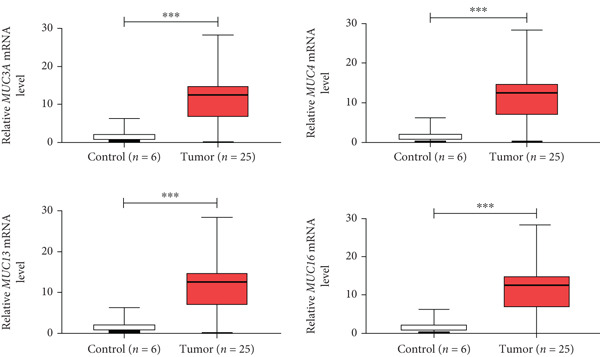
RT‐qPCR analysis of gene expression. The mRNA level of key *MUC* genes (a) *MUC3A*, (b) *MUC4*, (c) *MUC13*, and (d) *MUC16* is significantly ( ^∗∗∗^ *p* < 0.001) overexpressed in PDAC blood samples as compared to control blood samples.

In the present study, we have analyzed the expression level of key *MUC* genes in the Pakistani cohort comprising 25 blood samples from PDAC patients and 6 control blood samples. The RT‐qPCR analysis revealed that all target genes (*MUC3A*, *MUC4*, *MUC13*, and *MUC16*) are significantly overexpressed in tumor samples as compared to control samples. Statistical analysis was performed using Student’s *t*‐test, conforming to the statistical significance (*p* < 0.001) of the observed upregulation across target genes (Figure 5). These results underscore the differential expression pattern between tumor and control samples suggesting their relevance as signature biomarkers for cancer detection and progression.

## 4. Discussion

PDAC, the most prevalent subtype of pancreatic cancer, is the deadliest pathological condition, with a significant metastatic potential and a median survival rate of less than 1 year for patients in advanced disease stages [40]. The primary concern associated with this disease is a lack of early detection and effective approaches to treatment [7]. Poor prognosis, postsurgical recurrence, and complex biology aggravate the disease [7, 15].

A thorough comprehension of the aberrant expression patterns leading to PDAC may assist with closing the gap. In this regard, integrated high‐throughput sequencing and computational advances had provided an effective framework for complicated data analysis of open access cancer‐specific gene expression profiles to find possible biomarkers [41]. To achieve this, we investigated the potential of key *MUC* genes as diagnostic and prognostic biomarkers through a computational system biology approach. Moreover, to validate the findings of integrated computational analysis, the multiomics approach was employed.

Despite the fact that the survival data from our Pakistani cohort were not accessible, supportive Kaplan–Meier survival analysis employing TCGA pancreatic cancer dataset revealed that concurrent overexpression of *MUC3A*, *MUC4*, *MUC13*, and *MUC16* was significantly associated with poor DFS, with a hazard ratio (HR) of 1.8 and a log‐rank *p* value of 0.0097. This further supports our findings and suggests the potential of the key *MUC* gene signature as a prognostic biomarker in PDAC, underscoring the necessity of subsequent validation of these findings in a larger Pakistani cohort with longitudinal clinical follow‐up.

In the present study, to validate the findings at the in vitro level for accessing the practicability of the novel signature biomarker in biological systems, we have performed WES on the cohort from the Pakistani population to discover the mutational spectrum of tumor samples. Upon the bioinformatics analysis, a number of genetic variants were identified. The variants located within the coding region of the genome tend to have a greater impact on the proteins’ structure as well as function [42]. Hence, our research focused on assessing the impact of nonsynonymous mutations that have implication in pathology and determining the functional consequences of these mutations by predicting either neutral or disease‐causing effect of the mutation in specific genes.

Following that, comprehensive analysis of nonsynonymous mutations in key *MUC* genes was performed. To enhance the prediction accuracy, the mutations were first annotated by the following computational algorithms: Damagepredcount, SIFT, Polyphen2, MutationTaster, FATHMM, VEST4, PROVEAN, BAYESDel, ClinPred, and REVEL to characterize the pathogenic variant with neutral ones [43]. The presence of P1426H, S1576L, H2093Q, and S3319Y variants in the tandem repeat domain of *MUC4* can change the mucin interactome under normal and pathological conditions facilitating the interactions of mucins with the surface receptor to initiate oncogenic signaling leading to tumor proliferation, metastasis, and resistance to apoptosis which are characteristics of PDAC (Figure 2). The variants G1682X and S9301P are present in the amino terminal domain and T12180S, P13012L, H13241L, D13229H, W13569C, and W13257L in the tandem repeat domain of *MUC16* (Figure 3). *MUC16* plays a predominant role in PDAC by influencing tumor microenvironment that promotes tumorigenesis and metastasis [44]. Specifically, the interaction between *MUC16* and MSLN has been demonstrated to further aggravate PDAC by facilitating invasion and metastasis [45].

In the transcriptomic analysis, there were 2923 DEGs, with 768 genes being upregulated and 2155 genes being downregulated, and among the key *MUC* genes investigated, *MUC4* was the only one with statistically significant differential expression. The observed differential expression in only *MUC4* from the signature biomarker panel may be attributed to the sample size employed for transcriptomic analysis. The statistical power to identify differences in gene expression across the biomarker panels, with limited sample size, may be reduced, leading to the discovery of only *MUC4* with significant differential expression. Further research is required with a larger sample cohort to validate these expression patterns of key *MUC* genes under investigation.

Finally, RT‐qPCR was chosen to validate these findings as observed in high‐throughput screening methods due its high sensitivity, specificity, and quantitative accuracy. Our results demonstrate significant overexpression of key *MUC* genes in PDAC blood samples, with statistical significance  ^∗∗∗^ *p* < 0.001. The upregulation of key *MUC* genes in blood samples reinforces their potential as noninvasive biomarkers.

## 5. Conclusion

For improving the cancer diagnosis and management capabilities of the pathological condition, this signature biomarker (*MUC3A*/*MUC4*/*MUC13*/*MUC16*) plays a constructive role by enabling the real‐time monitoring through the noninvasive technique, paving the path for better patient outcomes through early diagnosis and targeted treatment methods. Therefore, it is imperative to tackle this aggressive malignancy by bringing these comprehensive investigations to the commercial level in order to make the practicality of early PDAC diagnosis.

**Nomenclature**
PDAC: pancreatic ductal adenocarcinoma
MAMs: membrane‐associated mucins
TCGA: The Cancer Genome Atlas
GTEx: Genotype‐Tissue Expression
WES: whole‐exome sequencing
RT‐qPCR: reverse transcription‐quantitative polymerase chain reaction
Hg38: human reference genome
BWA: Burrows–Wheeler Aligner
cDNA: complementary DNA

## Disclosure

All the authors read and approved the manuscript.

## Conflicts of Interest

The authors declare no conflicts of interest.

## Author Contributions

Maryam Naeem and Nimra Munir designed the project, performed analysis, and wrote the main manuscript. Maryam Naeem supervised the study and was responsible for the technical optimization of experimental procedures. Ibrar Ahmed helped in the analysis. Zarrin Basharat helped in proofreading, and Aneesa Sultan is the principal investigator of the lab where the research was conducted.

## Funding

Ibrar Ahmed was supported by a Brain Pool Fellowship Award by the National Research Foundation of Korea (RS‐2023‐00223245).

## Supporting Information

Additional supporting information can be found online in the Supporting Information section.

## Supporting information


**Supporting Information 1** (Significant.csv). List of significant differentially expressed genes identified from RNA‐seq analysis.

**Supporting Information 2** (S1APC‐2‐exonic.xlsx). Somatic exonic variants identified in mucins, including chromosomal position, reference and alternate alleles, functional annotation, and predicted impact in PDAC Sample 1.

**Supporting Information 3** (S7APC‐exonic.xlsx). Somatic exonic variants identified in mucins, including chromosomal position, reference and alternate alleles, functional annotation, and predicted impact in PDAC Sample 2.

**Supporting Information 4** (S8APC‐exonic.xlsx). Somatic exonic variants identified in mucins, including chromosomal position, reference and alternate alleles, functional annotation, and predicted impact in PDAC Sample 3.

## Data Availability

The data is publicly available after publication (https://submit.ncbi.nlm.nih.gov/sra/PRJNA1210086, submission ID: SUB15003800).
